# PHA-680626 Is an Effective Inhibitor of the Interaction between Aurora-A and N-Myc

**DOI:** 10.3390/ijms222313122

**Published:** 2021-12-04

**Authors:** Dalila Boi, Fani Souvalidou, Davide Capelli, Federica Polverino, Grazia Marini, Roberta Montanari, Giorgio Pochetti, Angela Tramonti, Roberto Contestabile, Daniela Trisciuoglio, Patrizia Carpinelli, Camilla Ascanelli, Catherine Lindon, Alessandro De Leo, Michele Saviano, Roberto Di Santo, Roberta Costi, Giulia Guarguaglini, Alessandro Paiardini

**Affiliations:** 1 Department of Biochemical Sciences, Sapienza University, 00185 Rome, Italy; dalila.boi@uniroma1.it (D.B.); fani.souvalidou@uniroma1.it (F.S.); federica.polverino@uniroma1.it (F.P.); grazia.marini@unimore.it (G.M.); roberto.contestabile@uniroma1.it (R.C.); 2 Institute of Crystallography, National Research Council, Monterotondo, 00015 Rome, Italy; davide.capelli@ic.cnr.it (D.C.); roberta.montanari@ic.cnr.it (R.M.); giorgio.pochetti@ic.cnr.it (G.P.); michele.saviano@ic.cnr.it (M.S.); 3 Institute of Molecular Biology and Pathology (IBPM), National Research Council, 00185 Rome, Italy; angela.tramonti@cnr.it (A.T.); daniela.trisciuoglio@uniroma1.it (D.T.); 4 Nerviano Medical Sciences S.r.l.–Oncology, Nerviano, 20014 Milan, Italy; patrizia.carpinelli@nervianoms.com; 5 Department of Pharmacology, University of Cambridge, Tennis Court Road, Cambridge CB2 1PD, UK; ca489@cam.ac.uk (C.A.); acl34@cam.ac.uk (C.L.); 6 Department of Drug Chemistry and Technologies, Pasteur Institut—Cenci Bolognetti Foundation, Sapienza University, 00185 Rome, Italy; alessandro.deleo@uniroma1.it (A.D.L.); roberto.disanto@uniroma1.it (R.D.S.); roberta.costi@uniroma1.it (R.C.)

**Keywords:** Aurora-A, N-Myc, neuroblastoma, PHA-680626, amphosteric inhibitors

## Abstract

Neuroblastoma is a severe childhood disease, accounting for ~10% of all infant cancers. The amplification of the MYCN gene, coding for the N-Myc transcription factor, is an essential marker correlated with tumor progression and poor prognosis. In neuroblastoma cells, the mitotic kinase Aurora-A (AURKA), also frequently overexpressed in cancer, prevents N-Myc degradation by directly binding to a highly conserved N-Myc region. As a result, elevated levels of N-Myc are observed. During recent years, it has been demonstrated that some ATP competitive inhibitors of AURKA also cause essential conformational changes in the structure of the activation loop of the kinase that prevents N-Myc binding, thus impairing the formation of the AURKA/N-Myc complex. In this study, starting from a screening of crystal structures of AURKA in complexes with known inhibitors, we identified additional compounds affecting the conformation of the kinase activation loop. We assessed the ability of such compounds to disrupt the interaction between AURKA and N-Myc in vitro, using Surface Plasmon Resonance competition assays, and in tumor cell lines overexpressing MYCN, by performing Proximity Ligation Assays. Finally, their effects on N-Myc cellular levels and cell viability were investigated. Our results identify PHA-680626 as an amphosteric inhibitor both in vitro and in MYCN overexpressing cell lines, thus expanding the repertoire of known conformational disrupting inhibitors of the AURKA/N-Myc complex and confirming that altering the conformation of the activation loop of AURKA with a small molecule is an effective strategy to destabilize the AURKA/N-Myc interaction in neuroblastoma cancer cells.

## 1. Introduction

AURKA, a member of the Aurora Kinases family, is a Ser/Thr kinase involved in the progression of mitosis. During the interphase, AURKA associates with centrosomes and controls their maturation in the G2 stage of the cell cycle, while in mitotic cells it localizes at spindle poles, where it plays a primary role in establishing spindle bipolarity and regulating microtubule nucleation and dynamic properties [1,2]. AURKA is widely overexpressed in a variety of solid tumors [3,4,5]. In particular, in neuroblastoma, a severe childhood cancer that arises from highly proliferative migratory cells of the neural crest [6], AURKA is highly expressed relative to normal tissues, and also displays a critical function by binding to and stabilizing the oncoprotein N-Myc [7]. 

Together with c-Myc and L-Myc, N-Myc constitutes a very potent network of transcription factors, regulating the expression of a massive group of genes implicated in cell cycle progression, protein translation, and metabolism [8,9]. Such transcription factors are usually found upregulated in various cancers in humans [10]. The structural organization of Myc proteins shares conserved motifs called “Myc Boxes” (MB), which serve as a docking site for protein–protein interactions. The turnover of Myc proteins is regulated by the phosphorylation of MBI (Myc Box I), which directs the protein towards ubiquitination and proteolysis [11]. N-Myc is primarily phosphorylated on Ser62 by the Cdk1/Cyclin-B complex, and then also on Thr58 by the Gsk3β kinase [12,13,14]. Dephosphorylation of Ser62 by the PP2A phosphatase recruits the activity of E3 ubiquitin ligase SCF^FBXW7^ [12,15,16]. The physical interaction of N-Myc with AURKA does not allow the ubiquitin ligase to intervene, thus preventing N-Myc degradation. The crystal structure of the complex between the purified kinase domain and the AURKA-interacting region of N-Myc (residues 61–89; Myc-AIR), which is the minimal motif required for AURKA binding, provided the structural basis of AURKA binding by N-Myc [17].

Over the years, serious efforts have been taken to exploit specific methods for targeting AURKA in cancer, mainly focused on the design of various inhibitors that are under investigation in clinical trials [18,19]. Apparently, an important subclass of these inhibitors, hailed as “conformation disrupting” (CD) or “amphosteric”, is also able to effectively disrupt the protein–protein interaction of the kinase with N-Myc, promoting the degradation of N-Myc itself [20,21,22]. The binding of such inhibitors induces a series of unique interactions that produce a conformational change on the structure of the kinase domain of AURKA, distinct from any known physiological state [23]. Notable examples come from CD532, MLN8054, or MLN8237 (Alisertib), diaminopyrimidine-derived compounds that disrupt the interaction between AURKA and N-Myc due to a significant opening of the N-terminal lobe of the kinase relative to the C-terminal one, and most importantly to the repositioning, i.e., “flip”, of the kinase activation loop (the “A-loop”, comprising residues 276–291 of human AURKA) in an inactive, hereinafter “closed”, conformation that prevents N-Myc binding [17,21].

Beside their classification as “orthosteric” (inhibiting kinase activity), “allosteric” (disrupting protein–protein interactions), or “amphosteric” (both), in general, kinase inhibitors are classified as “type I” or “type II”, according to their preference for a “DFG-out” or “DFG-in” target state, which refers in turn to the orientation of a catalytically important motif of three residues, i.e., Asp (D)-Phe (F)-Gly (G), found at the N-terminus of the kinase A-loop [24,25,26]. In the past, the closed state of the A-loop was invariably linked to the “DFG-out”-associated, type II inhibitors. However, recent studies have demonstrated that this is not a strict rule. For example, CD532, which is known to confer the closure of the A-loop, keeps the motif in the state “DFG-in”. Instead, a highly potent aminopyrimidinyl quinazoline AURKA type II inhibitor is not able to stabilize the A-loop in a closed state ([27]; PDB Code: 2C6E), as well as the potent MK8745 type-II compound, which does not act as a CD inhibitor [23,28].

Here we investigated, by computational and experimental means, which compounds already known to target AURKA at the ATP binding site (i.e., “orthosteric” compounds), regardless of their “type I” or “type II” nature, are able to act as CD inhibitors, and destabilize the complex with N-Myc. *In silico* data mining in the Protein Data Bank (PDB) of all known AURKA inhibitors, in a complex with the kinase domain of AURKA, was conducted to investigate which molecules are capable of opening the “angle” of the kinase (the shift of the N-terminal domain relative to the C-terminal one) in a similar way to other CD inhibitors, as well as to close the A-loop. A series of identified compounds potentially acting as CD inhibitors were firstly confirmed with Surface Plasmon Resonance (SPR) and kinase activity assays to bind to AURKA at the active site and to inhibit its enzymatic activity. Using competition assays with Myc-AIR in vitro and in cultured cells, we demonstrate that PHA-680626, so far known only as an inhibitor of the kinase activity of AURKA, is indeed able to also disrupt the AURKA/N-Myc complex. In conclusion, PHA-680626, together with CD532, MLN8054, and Alisertib, represents a new potential pharmacological tool to disrupt the “unholy matrimony” between two proteins that are mainly responsible for the severity and poor outcome of neuroblastoma.

## 2. Results

### 2.1. PHA-680626 and RPM1722 Are Predicted to Prevent N-Myc Binding to AURKA

**Figure 1:**
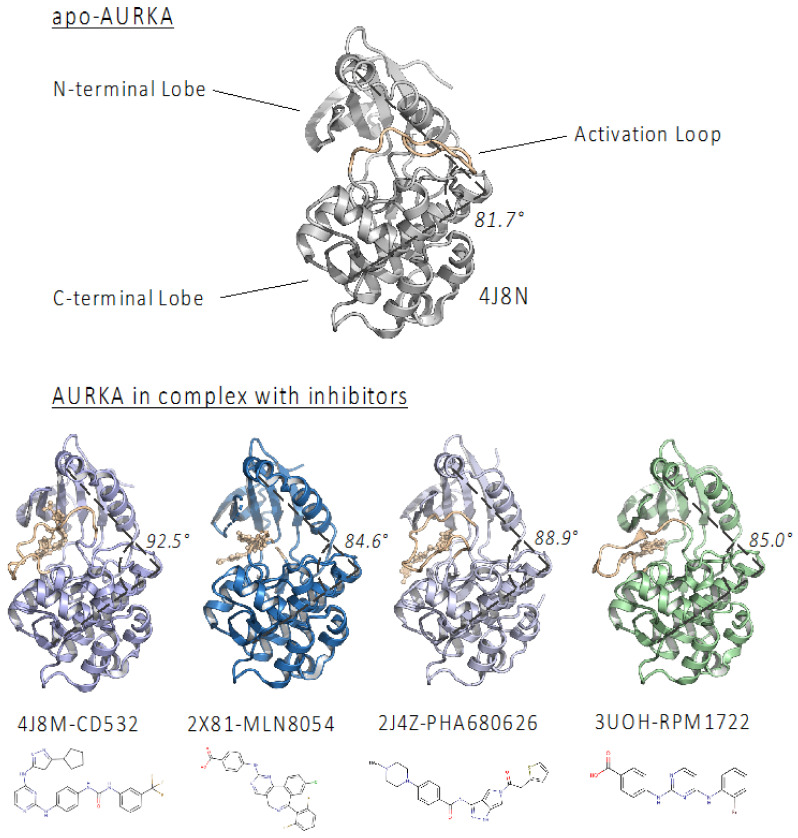
Structures of human AURKA catalytic domain in complex with the investigated inhibitors. The activation loop is shown in orange. The angle between the N- and C- lobes, measured between α-carbons of V324, E308, and A172, is also shown. The chemical structures of the compounds investigated are also represented.

In order to identify novel CD inhibitors of the AURKA/N-Myc complex, we started by screening the whole PDB subset of AURKA in complex with small molecule compounds (163 PDB entries). We then superposed each structure with apo-AURKA (PDB: 4J8N) and with the AURKA/N-Myc complex (PDB: 5G1X) [17], and identified four compounds (Figure 1 and Table 1) apparently acting as CD inhibitors, since they are able to (1) open the AURKA N-terminal domain relative to the C-terminal one (the opening was assessed by measuring the angle between the α-carbons of Val324, Glu308, and Ala172, as already described in [22]; (2) moving the A-loop of AURKA in a state that is incompatible with N-Myc binding. Among those, CD532 and MLN8054 were already shown to act as CD inhibitors [20,21]. Alisertib (MLN8237), whose structure in a complex with AURKA has not been solved yet, was also considered in this study for its very high similarity with MLN8054 and for its known CD activity [20,21]. Two additional compounds, PHA-680626 and RPM1722 [29,30], already known for being orthosteric inhibitors of AURKA, but not explored, to our knowledge, for their CD potential, were identified (Figure 1).

PHA-680626 derives from the optimization of PHA-680632, a compound based on a 1,4,5,6-tetrahydropyrrolo [3,4-c]pyrazole bis-cycle scaffold, which is known to target the ATP-binding pocket of kinases [31]. The 5-amidothiophene substituent of PHA-680626 is directed away from the glycine-rich loop and packs around Leu263 and Ala273, in the proximity of the DFG-motif. In an attempt to rationalize the ability of PHA-680626 to open the AURKA lobes and close the A-loop, we noticed a key interaction involving stacking between the thiophene ring of PHA-680626 and His280 of the A-loop.

RPM1722 relies on the bis-anilinopyrimidinic scaffold that is typical of several “DFG-in” inhibitors; however, a bromine substituent on the benzene ring of the scaffold, directed at the DFG flanking residue Ala273, is responsible for induced-dipole forces along the Ala273 side chain, which in turn are thought to induce the closing of the A-loop. This unique “DFG-out/loop-in” conformation is also stabilized by hydrogen bonding interactions between RPM1722 and residues Lys141 and His280, and by a conformational shift of Trp277, moving from a polar environment to a hydrophobic pocket [30].

These observations confirm that two other molecules, in addition to the already known CD inhibitors, are able to open the kinase lobes and close the A-loop, retaining AURKA in a conformation that could prevent N-Myc binding.

### 2.2. PHA-680626 and RPM1722 Behave as CD Inhibitors In Vitro

In vitro assays with CD532, MLN8054, PHA-680626, RPM1722, and Alisertib were initially carried out in order to measure their inhibitory activity on the purified kinase domain of AURKA (Table 1 and Supplementary Figure S1, see Supplementary Materials). Ten different concentrations of each compound were used in the presence of a concentration of ATP equal to the previously reported K_M_ [32]. The measured IC_50_ values were comparable to those found in the literature [21,30].

**Figure 2:**
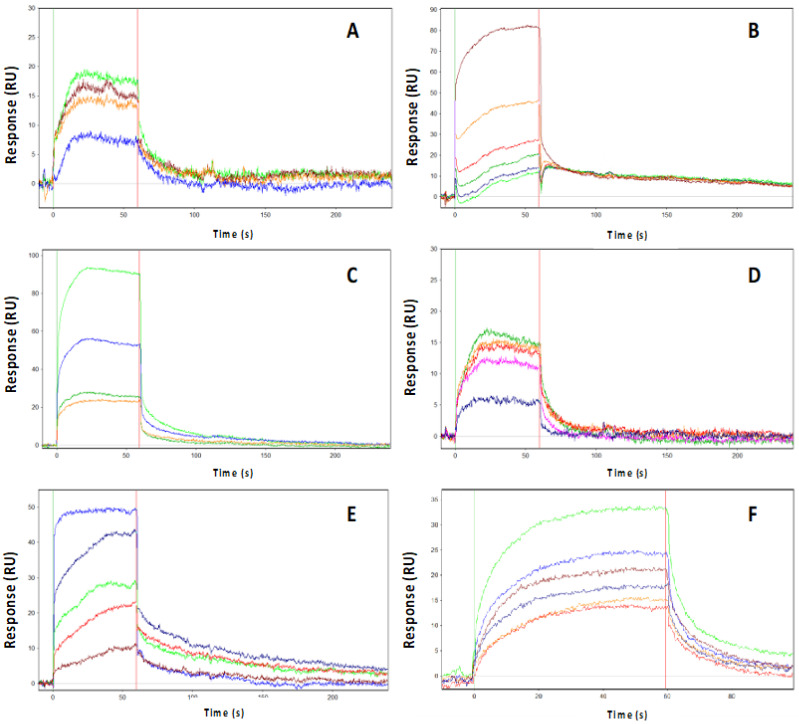
SPR analyses of ligands binding to AURKA. Full kinetic analysis of the AURKA inhibitors CD532 (**A**), MLN8054 (**B**), PHA-680626 (**C**), RPM1722 (**D**), Alisertib (**E**), and N-Myc (**F**). Determined binding parameters are listed in Table 2. Kinetic data for N-Myc: K_on_, 6.3 ± 1 × 10^4^ M^−1^s^−1^; K_off_, 0.0659 ± 0.0008 s^−1^; K_d_, 990 nM; Res SD 2.17. The interaction between AURKA and the ligands is indicated by the increase in Resonance Units (RUs) on the vertical axis compared to the baseline.

In addition, in order to further characterize the binding mode of the investigated inhibitors, their kinetic constants (K_on_, K_off_, and K_d_) were also evaluated using Surface Plasmon Resonance (SPR) binding assays (Table 2 and Figure 2). AURKA was immobilized onto a PCH sensor chip, and each compound was injected at increasing concentrations in the running buffer. The interaction between AURKA and the ligands was indicated by the increase in Resonance Units (RUs), compared to the baseline. Carrying out the experiments at a fast flow rate of 150 μL/min, which minimizes the effects of the dynamic A-loop conformation on the binding of the inhibitors, we obtained K_d_ values in good agreement with IC_50_ values for all compounds. An SPR binding kinetic assay was then used to evaluate the binding of Myc-AIR at the same conditions of flow rate and running buffer as for the inhibitors. A K_d_ value of ∼1 μM was obtained (Figure 2F).

**Figure 3:**
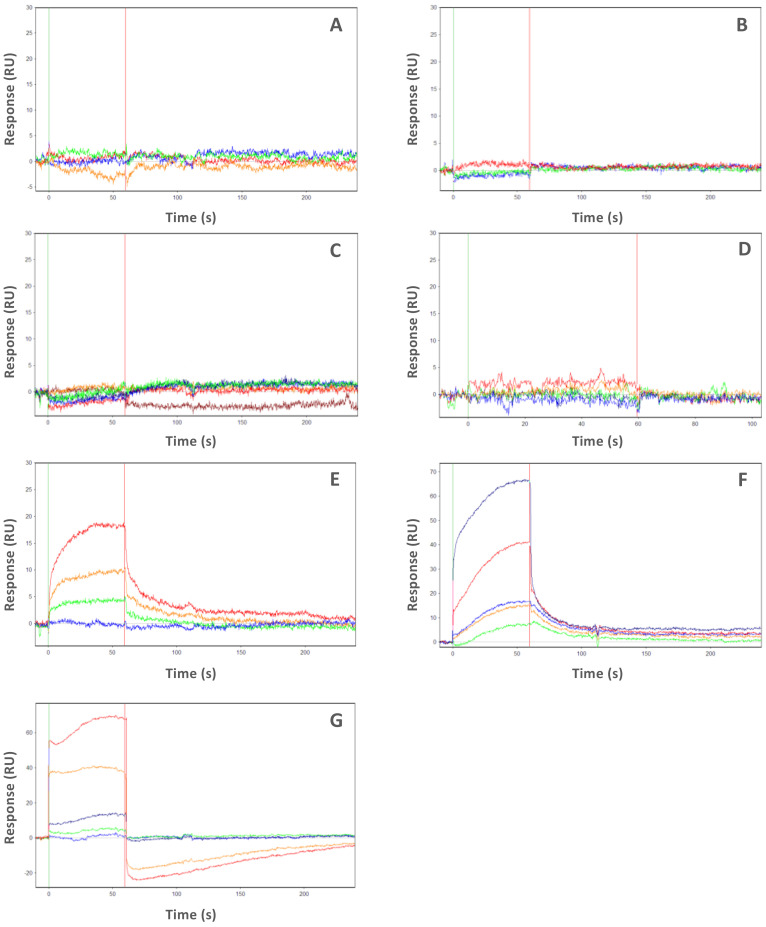
SPR analyses of N-Myc binding to AURKA in the presence of inhibitors. Sensorgrams of competition experiments between the inhibitors CD532 (**A**), MLN8054 (**B**), PHA-680626 (**C**), RPM1722 (**D**), Alisertib (**E**), MK8754 (**F**), ZM447439 (**G**), and N-Myc. The experiments were carried out by injecting Myc-AIR at different concentrations (125 nM, 250 nM, 500 nM, 1 μM, 2 μM, 4 μM, 8 μM, 16 μM) and at a constant flow rate (150 μL/min) of running buffer. Myc-AIR was previously diluted in running buffer containing a saturating concentration of the inhibitor (at least 10 times higher than its K_d_). Apparent K_d_ values of Myc-AIR are shown in Table 2. Apparent K_d_ values for the negative controls MK8754 and ZM447439 are 690 nM and 4 μM, respectively.

Then, in order to evaluate the ability of the inhibitors to interfere with Myc-AIR binding to AURKA, we performed SPR kinetic assays of Myc-AIR in the presence of a saturating concentration of each inhibitor (Figure 3). As a negative control, we also tested the orthosteric inhibitors MK8745 and ZM447439, which according to our structural investigation were not predicted as CD inhibitors. MK8745 (an analog of MK-5108 with IC_50_ of 0.6 nM) is one of the most potent and specific inhibitors of AURKA, while ZM447439 is a DFG-in, pan-Aurora family inhibitor, with a measured IC_50_ of ~100 nM [33,34]. Results in Table 2 indicate that the selected inhibitors almost completely prevent Myc-AIR from binding to AURKA (K_d_ of Myc-AIR > 15 μΜ or not measurable), while such an interaction remained unchanged upon competition with negative control compounds. Thus, SPR data confirmed the behavior of the investigated inhibitors as CD compounds, substantiating our initial hypothesis.

### 2.3. PHA-680626 Behaves as a CD Inhibitor in MYCN Overexpressing Cell Lines

**Figure 4:**
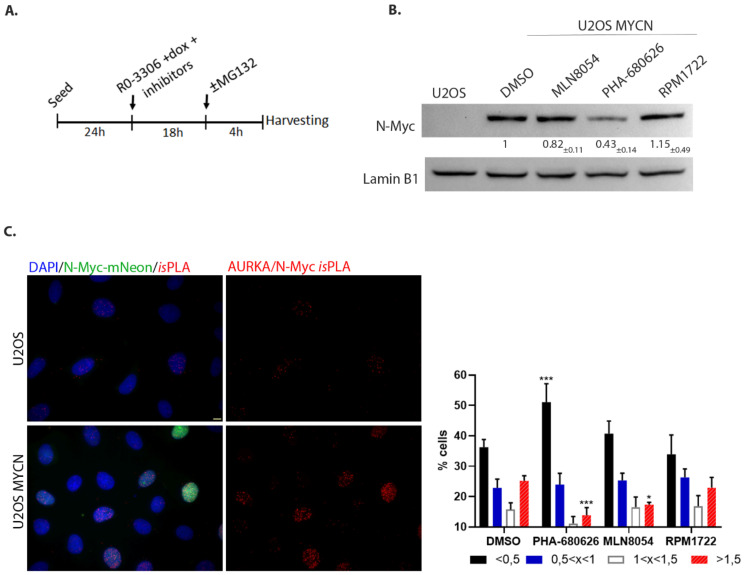
PHA-680626 is able to decrease AURKA/N-Myc interaction. (**A**) Schematization of the combined synchronization and treatment protocol. (**B**) Western blot analysis from U2OS and U2OS MYCN cell lines treated with 1 μM of each compound, or DMSO as control, following the protocol described in (**A**), without MG-132. Lamin B1 was used as loading control. The mean and SD of the quantification of N-Myc signal from three independent experiments are indicated. (**C**) in situ Proximity Ligation Assays to visualize the AURKA/N-Myc complex formation. The fluorescence panels show examples of the negative (U2OS that do not express N-Myc) and the positive (U2OS MYC dox-induced) reference cultures. The histograms on the right show the distribution (%) of cells in classes, defined by *is*PLA spot values. Values represent *is*PLA spots per nucleus, normalized to the average value in control cells (at least 1400 measured cells per condition, from four independent experiments); mean and SDare shown. Statistical analysis by 2-way ANOVA-Tukey’s multiple comparison, *: *p* < 0.05; ***: *p* < 0.001. Scale bar: 10 µm.

We then wanted to test whether PHA-680626 and RPM1722 were able to also inhibit the N-Myc/AURKA interaction in cultured cells. A U2OS cell line that does not express endogenous N-Myc but is engineered for inducible doxycycline-dependent expression of mNeon-tagged N-Myc was synchronized in the late G2 phase, when AURKA levels are highest, by the RO-3306 Cdk1 inhibitor (protocol in Figure 4A; Supplementary Figure S2A). Interestingly, in cultures treated with 1 μM of each compound, N-Myc failed to accumulate to levels observed in control cultures (DMSO) after treatment with PHA-680626 (Figure 4B). This result suggests an impaired interaction of AURKA with N-Myc, and the subsequent destabilization of the latter. Therefore, to directly measure the interaction between AURKA and N-Myc under these conditions, in situ Proximity Ligation Assay (*is*PLA) reactions were performed, adding the proteasome inhibitor MG-132 4 h before fixation, to counteract potential destabilization effects (Figure 4C and Supplementary Figure S2B). After treatment with the investigated inhibitors, the raw number of *is*PLA spots within the cell nuclei was normalized by the mean value of the control population. Cells were then assigned to four different classes, based on their number of normalized spots per nucleus (Figure 4C). In MLN8054-treated cultures, the fraction of cells with the highest normalized number of *is*PLA interaction spots (red columns) was significantly reduced, indicating an impaired AURKA/N-Myc interaction. Notably, this effect was even more pronounced after PHA-680626 treatment and paired with a strong increase in the percentage of cells with the lowest *is*PLA signals (black columns). Surprisingly, and in contrast with in vitro data, RPM1722 seemed quite ineffective. To confirm that the interaction impairment that we observed was produced by the CD ability of PHA-680626 to modify the AURKA lobe orientation and A-loop conformation, we repeated the experiment with the orthosteric AURKA inhibitor ZM447439. The results show no difference between control and treated cells (Supplementary Figure S3A), supporting our initial assumption.

**Figure 5:**
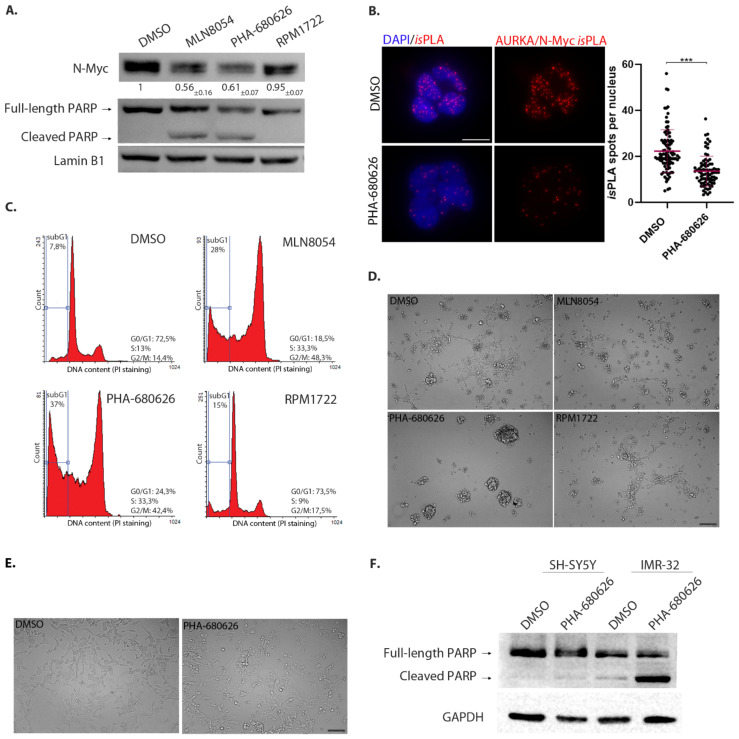
PHA-680626 causes N-Myc protein decrease and cellular stress in neuroblastoma cells. (**A**) Western blot analysis from IMR-32 neuroblastoma cell line treated with 1 μM of each inhibitor, or DMSO for control, for 48 h. Both full-length and cleaved PARP-1 signals are shown, as indicated. The mean and SD of the quantification of N-Myc signal from three independent experiments are indicated. (**B**) In situ Proximity Ligation Assay of AURKA/N-Myc complex in IMR-32 cells treated with 1 μM PHA-680626 and MG-132 for 4 h. The graph shows the number of spots of interactions per nucleus from three independent experiments (at least 207 cells per condition were analyzed). Statistical analysis by Mann Whitney test, *** *p* < 0.0001, scale bar 10 μm. (**C**) FACS analysis of IMR-32 cells treated as in (**A**) and stained with PI. The region used to evaluate the fraction of cells displaying sub-G1 DNA content is indicated. The percentage of vital cells distributed in each phase of the cell cycle, as well as the percentage of cells with sub-G1 DNA content calculated on the total acquired events, are shown (one experiment representative of three independent experiments). (**D**) Morphological appearance of IMR-32 cultures by bright-field microscopy (20× magnification), treated as in (**A**). Scale bar: 100 μm (**E**) Morphological appearance of SH-SY5Y neuroblastoma cultures by bright-field microscopy (20× magnification), treated as in (**A**). Scale bar: 100 μm. (**F**) Western blot analysis from SH-SY5Y and IMR-32 cells treated as in (**A**).

We then moved to a neuroblastoma MYCN-amplified cell line, IMR-32. In this case, treatment for 48 h with 1 μM PHA-680626 decreased N-Myc levels in a comparable manner to MLN8054; again, no major effect was observed with RPM1722 treatment (Figure 5A). Consistently, PHA-680626 treatment yielded a significant reduction in the number of AURKA/N-Myc interaction *is*PLA signals in interphase nuclei (Figure 5B). By FACS analysis, PHA-680626 treatment resulted in an increase in the fraction of cells with G2/M DNA content, similar to known AURKA inhibitors (e.g., MLN8054, Figure 5C), while RPM1722 did not induce any major cell cycle change. In addition, PHA-680626- and MLN8054-treated cultures displayed a fraction of cells with sub-G1 DNA content (about 40% and 30%, respectively, compared to 8% and 15% in DMSO or RPM1722 conditions), as well as a fraction of cleaved PARP-1 in Western blot analysis (Figure 5A,C), indicative of cell death induction. Interestingly, in contrast to RPM1722, PHA-680626 administration affected IMR-32 cells morphology (Figure 5D), with the disappearance of viable substrate-adherent cells and the formation of cellular aggregates that detached from the surface. As expected, the administration of ZM447439 neither led to N-Myc protein degradation, nor did it cause evident morphological features associated with cytotoxicity, but rather only induced spherical mitotic shapes, consistent with delayed progression in mitosis (Supplementary Figure S3B,C).

Surprised by the observed lack of activity of RPM1722, we assessed the ability of the compound to bind and inhibit AURKA in cultured cells. To this aim, we quantified the fluorescence intensity of pT288 AURKA, a marker of AURKA kinase activity, after treatment with 1 µM RPM1722 for 24 h (Supplementary Figure S3D). As evidenced by the fluorescence intensity signal of pT288 AURKA, the capability of RPM1722 to inhibit AURKA was indeed lower compared to MLN8054, suggesting a reduced ability of the former to reach its cellular target.

We then asked whether PHA-680626 treatment differentially affects MYCN amplified versus MYCN non-amplified neuroblastoma cells. To this aim, we used PHA-680626 in the SH-SY5Y cell line, which does not display MYCN amplification, under the same conditions employed in IMR-32 cells. Interestingly, no changes in the cell morphology (Figure 5E) compared to DMSO-treated cells were evident. In addition, we did not observe cell death induction as assessed by PARP-1 cleavage (Figure 5F) or Annexin V/PI staining (Supplementary Figure S3E), indicating differential sensitivity of neuroblastoma cell lines, depending on MYCN amplification status. Together, these results suggest that PHA-680626 is a strong and effective CD inhibitor, preventing the AURKA/N-Myc interaction in cultured cells, triggering N-Myc degradation, and impairing the viability of neuroblastoma cell lines that are N-Myc-addicted for their proliferation.

## 3. Discussion

Poor prognosis for neuroblastoma patients is associated with elevated levels of N-Myc, a transcription factor favoring cell cycle progression, cellular proliferation, and metastasis [9,35]. In spite of the paramount importance of this oncoprotein, the very low druggability of N-Myc has so far prevented any successful strategy for chemotherapeutic intervention. However, recent studies demonstrating the physical interaction between the mitotic kinase AURKA and N-Myc sequesters the latter from proteolytic degradation [7,17], paved the way for a novel, promising chemotherapeutic approach in neuroblastoma. These findings have prompted the identification of compounds able to disrupt the AURKA/N-Myc complex, in order to promote the degradation of the latter. Already known ATP-competitive inhibitors of AURKA, targeting the kinase active site, were demonstrated to also inhibit such an interaction. Among these, CD532 is the best characterized CD inhibitor so far, as it is able to alter the conformation of AURKA in a way that almost completely prevents N-Myc binding [21,22]. However, the observation that CD532 lacks drug-like properties, due to its short half-life and poor oral bioavailability [21], urges the need to identify new small-molecule disruptors of the AURKA/N-Myc interaction.

As the amphosteric effects are neglected in most current inhibitor screenings [21], we investigated already known AURKA inhibitors with the aim of (1) expanding the repertoire of amphosteric compounds, and (2) at the same time, providing additional insights for their observed antineoplastic effects. To this end, we started this study from structural data mining on PDB, to identify crystal structures of AURKA whose conformation, due to the complex with active site inhibitors, was unsuited for N-Myc binding. The comparison of such AURKA structures with apo-AURKA (PDB: 4J8N) showed that the N- and C-terminal lobes of the kinase domain can adopt a very diverse set of orientations, ranging from ~85° in the case of MLN8054, to ~93° for CD532, and different conformations of the A-loop in the closed state, confirming that the view of kinases existing mainly in two states, “active” or “inactive”, based on the DFG-motif orientation (DFG-in -> active, DFG-out -> inactive) is somewhat simplistic. For example, CD532, the best-characterized amphosteric inhibitor so far, and the one exerting at the same time the amplest opening of the AURKA lobes and the most dramatic effects on N-Myc degradation, is paradoxically also a “DFG-in” inhibitor, according to the position of the DFG motif [22].

Those compounds that were predicted to disrupt the AURKA conformation, in a way similar to MLN8054 and CD532, were obtained and further characterized. A series of initial kinase activity assays confirmed the inhibitory ability of each compound against AURKA, with IC_50_ in close proximity to the ones reported in the literature with different in vitro/in vivo assays [21,30,36]. Full kinetic analysis of the AURKA inhibitors by SPR confirmed that MLN8054, with a one-digit nanomolar K_d_ and IC_50_ values, was the most potent AURKA inhibitor tested, and it was therefore adopted as an archetypal amphosteric inhibitor in the next comparative analyses. 

**Figure 6:**
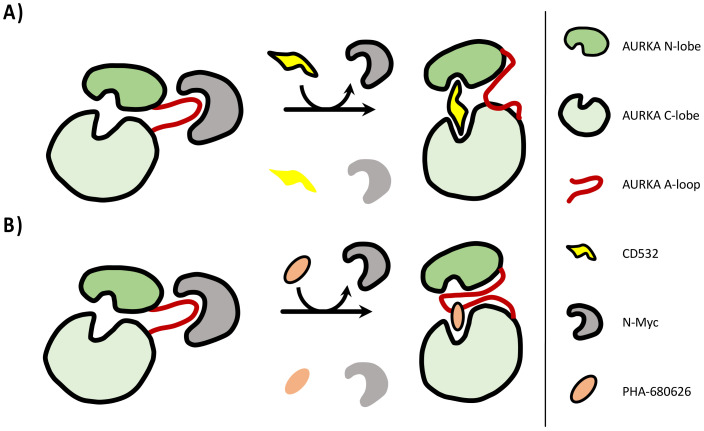
Scheme of the conformational disruption of AURKA/N-Myc interaction. CD inhibitors can dissociate N-Myc by adopting two distinct strategies: wide-opening the N-terminal lobe of the kinase to create an ideal cleft for A-loop closure or stabilizing the A-loop in a closed conformation via formation of direct contacts. (**A**) CD532 does not engage any stabilizing contact with the A-loop. Instead, the bulky moiety of CD532 wide-opens the N-terminal lobe of the kinase, creating a large cleft in which the A-loop is stabilized in an inactive orientation. (**B**) The thiophene moiety of PHA-680626 is able to stabilize the A-loop in a close conformation that is unproductive for N-Myc binding, thanks to a stacking interaction with His280 of the A-loop.

A straightforward kinetic characterization of protein–ligand interactions was attained using SPR, confirming that RPM1722 and PHA-680626 were indeed able to interfere with the AURKA/N-Myc complex formation. In one case (i.e., Alisertib), where it was still possible to measure an apparent K_d_ Myc-AIR, the observed effect was to lower the affinity of Myc-AIR for the kinase. All the other investigated compounds were able to completely inhibit the interaction at saturating concentrations (10 times higher than their K_d_). Notably, PHA-680626 was responsible for complete dissociation of the complex. Closer inspection of the crystal structure and a detailed comparison of PHA-680626 with the highly similar PHA-739358 (Danusertib), the structure of which was solved in complex with AURKA (PDB 2J50) [29], provided a rationale to the different behavior of the two closely related compounds (Supplementary Figure S4A). Indeed, the thiophene moiety of PHA-680626 is able to stabilize the A-loop in a close conformation that is unproductive for N-Myc binding, thanks to a stacking interaction with His280 of the A-loop. Conversely, the bulkier methoxy moiety of Danusertib hampers such interaction and prevents the A-loop of AURKA to adopt a “closed” conformation. Of note, topologically similar hydrophobic interactions were also observed in the structure of AURKA/MLN8054 and predicted via modeling (Supplementary Figure S4B) in the case of the fluorophenyl moiety of Alisertib (residues Val147, Ser278 and Val279), thus suggesting such interaction as an important pharmacophore feature for stabilizing the closed conformation of the A-loop, and conferring an amphosteric behavior to the inhibitor. It is interesting to note that CD532 achieves a similar conformational change of the A-loop, without engaging any stabilizing contact with the latter. Indeed, the deeply buried 3-trifluoromethyl-biphenyl urea moiety of CD532 induces this inactive conformation via displacement and interactions with the β1 and β2 strands of the N-terminal lobe of AURKA, without reorienting the DFG motif, but in this way wide-opening the N-terminal lobe of the kinase, creating a large cleft in which the A-loop is stabilized in an inactive orientation through a unique network of hydrogen bonds. Therefore, taken together with structural data, these observations suggest the conformational disruption of AURKA that is necessary to dissociate N-Myc can be achieved by adopting two complementary, but distinct strategies: stabilizing the A-loop in a closed conformation via formation of direct contacts, and/or wide-opening the N-terminal lobe of the kinase to create an ideal cleft for A-loop closure (Figure 6).

It is important to note that, in any case, fairly high concentrations of inhibitors were necessary to hamper the formation of the AURKA/N-Myc complex in cells. Such high concentrations, which are necessary to unveil the amphosteric potential of AURKA inhibitors, suggest the difficulty that small inhibitors encounter when competing with the AIR of N-Myc. It was previously shown that AURKA is in dynamic equilibrium between active and inactive A-loop conformations and that the position of this equilibrium can be shifted by the binding of other ligands [37]. The kinetics of N-Myc binding to AURKA (Figure 2F) measured by means of SPR suggests that an initial low association rate (K_on_) is then followed by a quite stable complex that shifts the dynamic equilibrium of AURKA in a conformation disfavoring successive binding of CD inhibitors. In this scenario, our data suggest that an amphosteric inhibitor of AURKA with a very low dissociation rate (in our study, PHA-680626, for example, is the one with the lowest K_off_), which is independent of the concentration of the compound, might be more effective in displacing N-Myc. This observation, together with the observed inability to reach and inhibit AURKA in cultured cells, could account for the lack of the amphosteric behavior of RPM1722. It is therefore conceivable that further optimization of this scaffold would be required, involving the improvement of cell permeability, by rational-based modifications of the original scaffold (e.g., carrier-linked prodrugs).

Recently, it has been shown that covalent Coenzyme A modification of AURKA via binding to the Cys290 residue of the A-loop is specific and provides the proof of concept for a potential “dual anchor” irreversible inhibitory mechanism of AURKA [38]. Such suicide inhibition would show the lowest reachable K_off_ value and would possibly keep the A-loop of AURKA in a conformation unsuitable for N-Myc binding, relieving it from the need for high inhibitor concentrations. In this direction, our study expands with PHA-680626 the repertoire of AURKA/N-Myc amphosteric scaffolds, to start from for further development of small molecules targeting MYCN-amplified malignancies.

## 4. Materials and Methods

### 4.1. Screening on Protein Data Bank and Homology Modeling

CD532 (PDB entry: 4J8M) and MLN8054 (PDB entry: 2X81) were used as a prototype of amphosteric inhibitors to quantitatively measure the magnitude of the conformational disrupting effect, as suggested in [22]. The opening between the N-terminal domain compared to the C-terminal lobe was asserted by measuring the dihedral angle between the α-carbons of V324, E308, and A172, comparing it to the one measured for apo-AURKA (empty binding pocket, 4J8N, angle: 81.7°) [21]. In order to generate the ensemble of crystallographic structures for our analysis, we conducted data mining of all the available entries of the AURKA kinase on the (PDB). We have identified 198 structures for the AURKA kinase overall and, in 95 of them, the kinase in a complex with already known orthosteric inhibitors. Subsequently, we conducted a visual examination of the identified structures using the open-source molecular visualization system PyMOL (The PyMOL Molecular Graphics System, Version 1.2 r3pre, Schrödinger, LLC, Cambridge, UK).

We proceeded by aligning every protein–inhibitor complex with the PDB entries 4J8M and 2X81 in order to find other molecules that can both open the “angle” of AURKA and induce a complete flip of the activation loop, retaining it in a “closed” conformation not able to bind N-Myc. We excluded from our examination all the structures that lacked resolved residues at the key N-Myc binding regions; proceeding this way, we selected 10 entries (PDB codes: 2J4Z, 2J50, 2X6E, 2X81, 2WTV, 3UOJ, 3UOH, 3UNZ, 3UO6, 4J8M, 5ONE). We then conducted another screening to exclude the “doublet” entries, in which AURKA is in a complex with the same molecule (e.g., 2X81 and 2WTV in a complex with MLN8054) and to consider only the best candidate in a family of inhibitors with the same backbone and the same structural change (e.g., 3UO6, 3UOJ, 3UOH, 3UNZ have the same chemical scaffold, but RPM1722 (3UOH) has the best affinity for AURKA, with a K_d_ = 13 ± 2.2 nM). The final dataset was formed by four structures: 4J8M [21], 2X81 [39], 2J4Z [29], and 3UOH [30]. The crystal structure of AURKA in a complex with MLN8054 (PDB: 2X81) was used as a starting point to generate the model of AURKA in a complex with Alisertib, using the “homology modeling” tool of PyMod 3.0 [40].

### 4.2. Purification of AURKA Catalytic Domain

For the purification of the catalytic domain of human AURKA (amino acids 122–430), the plasmid pETM11 was used. The resulting protein contains an N-terminal His-tag and an intervening TEV protease cleavage site. For the pre-inoculation, more colonies were taken from a plate and resuspended in LB containing 40 μg/mL kanamycin, with growth at 37 °C overnight. After inoculation 1:100 in 2 L of LB, the culture was grown at 37 °C for 4 h and 30 min. When OD 600 nm achieved the value of 0.5, the expression of the recombinant protein was induced with 0.2 mM IPTG, and the culture was incubated at 28 °C overnight. The pellet, after being centrifuged at 5500 rpm, at 4 °C for 20 min, was resuspended in about 130 mL of 50 mM Hepes, pH 7.4, 5 mM MgCl_2_, 300 mM NaCl, 10% glycerol (buffer A), and 1 complete Protease Inhibitor Cocktail tablet (SIGMA-Aldrich, St. Louis, MO, USA), sonicated in ice (for 2 min, 20 sec pulse on, and 20 sec pulse off), and centrifuged at 12,000 rpm for 25 min at 4 °C, twice. The obtained supernatant was filtered and loaded onto a 5 mL HisTrap column (GE Healthcare, Chicago, IL, USA), previously equilibrated with buffer A (flow rate 1 mL/min). The column was washed with 10 mL buffer A and then with buffer A containing 20 mM, 40 mM, and 100 mM imidazole. Elution was performed with buffer A + 300 mM imidazole. Fractions containing AURKA were collected and dialyzed against Buffer A, at 4 °C. In order to use the kinase for the activity assays, it was essential to phosphorylate the purified catalytic domain, so as to activate it. That was accomplished by incubation of AURKA with 400 μΜ of ATP (c > 10fold of K_M_) for 3 h on ice. To remove the ATP in excess, the protein was dialyzed in Buffer A overnight at 4 °C. Once activated, AURKA was aliquoted and stored at −80 °C, without losing stability or activity.

### 4.3. Chemistry

Compounds for testing were purchased from Molport (Riga, Latvia, https://www.molport.com/). All chemicals were of the highest purity available (guaranteed purity of over 90% by H-NMR or LCMS), as certified by the vendor. PHA-680626 was a gift from Nerviano Medical Sciences Srl (Milan, Italy). The synthesis of RPM1722 (RDS4007) was carried out as reported in Scheme S1 (see Supplementary Information), using the 2,4-dichloropyrimidine as the starting material. Two subsequent aromatic nucleophilic substitutions were performed. First, the 2-bromoaniline was introduced in position 4 of the pyrimidine ring in the presence of aqueous hydrochloric acid (1.0 M), allowing the reaction to proceed for 36 h at room temperature. Then, the second chlorine atom was substituted with 4-aminobenzoic acid, by means of a microwave-assisted reaction performed using absolute ethanol as a solvent at 100 °C for 20 min. The synthesis was carried out providing some changes to a previously published work [41]. The synthetic scheme, the procedures, and the analytical spectroscopic data are reported in the Supplementary Information.

### 4.4. Kinase Activity Assays

All kinase assays for the AURKA catalytic domain were performed with the ADP-Glo™ Kinase Assay Kit (Promega, Madison, WI, USA). For Alisertib, the concentrations tested were: 0.075 nM, 0.155 nM, 0.312 nM, 0.665 nM, 1.250 nM, 2.5 nM, 5 nM, 10 nM, 20 nM, and 40 nM; for CD532: 3.12 nM, 6.25 nM, 12.5 nM, 25 nM, 50 nM, 100 nM, 200 nM, 400 nM, 800 nM, and 1600 nM; for MLN8054: 0.15 nM, 0.31 nM, 0.62 nM, 1.25 nM, 2.5 nM, 5 nM, 10 nM, 20 nM, 40 nM, 80 nM, and 160 nM, for PHA-680626: 3.12 nM, 6.25 nM, 12.5 nM, 25 nM, 50 nM, 100 nM, 200 nM, 400 nM, 800 nM, and 1600 nM; for RPM1722: 0.5 nM, 1 nM, 2 nM, 4 nM, 8 nM, 16 nM, 32 nM, and 64 nM. The reaction mixture (20 μL) contained the different concentrations of each inhibitor (obtained by adding 1μL of inhibitor solubilized in 100% DMSO), 35 μM ATP, 100 ng AURKA in 16 mM Tris HCl, pH 7.5, 8 mM MgCl_2_, 0.04 mg/mL BSA, 0.2 mM DTT. After incubation at room temperature for 30 min, the reaction was started by adding 5 μL of the Myelin Basic Protein (20 mg/mL), a substrate of AURKA, and was incubated at 30 °C. Then, the assay was performed in two steps; first, an equal volume (25 μL) of the ADP-Glo™ Reagent was added to terminate the kinase reaction and deplete the remaining ATP. Second, the Kinase Detection Reagent (50 μL) was added to simultaneously convert ADP to ATP and allow the newly synthesized ATP to be measured using a luciferase/luciferin reaction. The luminescence produced at the end of every reaction, which corresponds to the kinase activity of AURKA, was measured as a relative light unit (RLU) with a Luminoskan™ Microplate Luminometer (Thermo Fisher Scientific, Waltham, MA, USA). Data analysis was carried out with the GraphPad Prism 8 software, using the following equation:

RLU = 100 × (1 − [I]^n/([I]^n + 〖IC50〗^n)) + const

from what we obtained the IC_50_, with [I] as the concentration of inhibitor, n the Hill coefficient, and const as residual activity.

### 4.5. Surface Plasmon Resonance

SPR experiments were performed at 25 °C using a Pioneer AE optical biosensor (Molecular Devices-ForteBio, Fremont, CA, USA) equipped with a PCH sensor chip and equilibrated with running buffer 10 mM Hepes pH 7.4, 150 mM NaCl, 1% DMSO, 0.005% Tween20. The sensor chip was chemically activated for 7 min by injecting 175 μL of a 1:1 mixture of 100 mM N-hydroxysuccinimide (NHS) and 400 mM ethyl−3 (3-dimethylamino) propyl carbodiimide (EDC) at a flow rate of 25 μL/min. AURKA was immobilized on both Ch1 and Ch3 channels of the activated sensor chip by using standard amine-coupling procedures [42]. Flow cell Ch2 was left empty and used as a control. Briefly, a 0.1 mg/mL AURKA solution (in 10 mM sodium acetate, pH 4.5) was injected at 10 μL/min on channels 1 and 3 (channel 2 was used as reference), followed by a 70 μL injection of 1 M ethanolamine pH 8.0 to block any remaining activated groups on the surface. AURKA was captured to a density of ~14,000 resonance units (RUs) on both Ch1 and Ch3 flow cells. The stability of the AURKA surface was demonstrated by the flat baseline achieved at the beginning (0–60 s) of each sensorgram. To correct for bulk refractive index shifts, a DMSO calibration plot was also constructed (buffer sample containing 0.5–1.5% DMSO) [43]. 

Compounds for testing were solubilized in 100% DMSO and then diluted in 10 mM Hepes pH 7.4, 150 mM sodium chloride and 0.005% Tween20 up to a final concentration of 1% DMSO (running buffer) or 4% (CD532) and injected at different concentrations onto the sensor chip at a constant flow rate of 150 μL/min. A dissociation of 180 s was used and a mild regeneration of the surfaces was carried out every three injections using a solution of 1 M NaCl and 10 mM NaOH. At least two experiments for each analyte were performed. The interaction of the tested inhibitors with immobilized AURKA was detected as a measure of the change in mass concentration, expressed in RUs. The same conditions were used for the competition experiments between the compounds and Myc-AIR, respectively. In this case, Myc-AIR (custom peptide from GenScript, Leden, Netherlands) was diluted in running buffer containing the inhibitor at a constant concentration 10 times higher than its K_d_. Different concentrations of Myc-AIR (ranging from 125 nM to 16 μM) were then injected into the sensor chip at a constant flow of 150 μL/min of the running buffer containing the inhibitor at a saturating concentration. All sensorgrams were processed using double referencing [44]. First, responses from the reference surface (Ch2) were subtracted from the binding responses collected over the reaction surfaces to correct for bulk refractive index changes between the flow buffer and analyte sample. Second, the response from an average of the blank injections (zero analyte concentrations) was subtracted to compensate for drift and small differences between the active channel and the reference flow cell Ch2 [45]. To obtain kinetic rate constants and affinity constants, the corrected response data were fit in the QDAT software. Kinetic analysis of each ligand/analyte interaction was obtained by fitting the response data to a reversible 1:1 bimolecular interaction model. The equilibrium dissociation constant (K_d_) was determined by the ratio k_off_/k_on_.

### 4.6. Cell Lines, Synchronization Protocols and Treatments

Cell lines were grown at 37 °C and 5% CO_2_. For the generation of the human U2OS/MYCN-tFT osteosarcoma cell line, N-Myc (#74163, Addgene, Watertown, MA, USA) was cloned into plasmid pcDNA5/FRT/TO (Thermo Fisher Scientific) bearing an mCherry and mNeon tandem fluorescent tag (tFT, [46]; kind gift of Michael Knop, ZMBH, University of Heidelberg). This construct was used to create the N-Myc-tFT cell line by Flp recombinase-mediated integration into the genome of a U2OS FRT/TO Flp-In host cell line (kind gift of Adrian Saurin, Dundee) according to the manufacturer’s instructions for the Flp-InTM T-RExTM System (Thermo Fisher Scientific). This cell line was grown in complete DMEM (Dulbecco’s Modified Eagle Medium) supplemented with 10% tetracycline-free fetal bovine serum (FBS), and the induction of N-Myc expression was obtained by adding 1 µg/mL doxycycline (tetracycline analogue; Santa Cruz Biotechnology, Dallas, TX, USA). Treatment with 9 µM Ro-3306 (Sigma-Aldrich, SML0569) for 22 h or with 10 µM MG-132 (Chem-Cruz, sc-201270) for 4 h was performed when indicated. The IMR-32 and SH-SY5Y neuroblastoma cell lines (kind gift from Prof. Giuseppe Giannini, Sapienza University of Rome) were grown in MEM (Minimum Essential Medium) containing 1% non-essential amino acids or in DMEM F12, respectively, both supplemented with 10% FBS. Each cell line was treated with 1 µM of inhibitors (MLN8054, PHA-680626, RPM1722, ZM447439), or 0.1% DMSO as a control, for 4 h, 22 h, or 48 h, as indicated. Images of IMR-32 and SH-SY5Y cultures after treatments were acquired using a ZOE fluorescent cell imager (Biorad, Hercules, CA, USA).

### 4.7. In Situ Proximity Ligation Assays (isPLA)

*In situ* proximity ligation assays (*is*PLA) were performed on cells grown on coverslips and fixed with 3.7% formaldehyde/30 mM sucrose in PBS, for 10 min at room temperature, followed by permeabilization in PBS containing 0.1% TritonX-100, 5 min at room temperature. The Duolink PLA kit (DUO92007, Sigma-Aldrich) was used according to the manufacturer’s instructions. The amplification time was 100 min and the primary antibodies pair used to detect the interaction was rabbit anti-Aurora-A (14475, Cell Signaling, 1:100 o.n., Danvers, MA, USA) and mouse anti-N-Myc (NCM II 100, 1:20, Calbiochem). Counterstaining with DAPI (DUO82040, DAPI mounting media, Sigma Aldrich) was performed at the end of the procedure. For quantification of *is*PLA fluorescence signals, samples were analyzed with an inverted microscope Eclipse Ti (Nikon) using a Clara camera (ANDOR technology) and 60× (oil immersion, N.A. 1.4) objective along the *z*-axis every 0.4 μm for a range of 3.2 μm (for the U2OS MYCN cell line) or 5 μm (for the IMR-32 cell line). Images were acquired in the automated mode with the JOBS module of the Nis-Elements H.C. 5.11 software. The “general analysis” module of Nis Elements H.C. 5.11 was then used for an automatic spot count; *is*PLA signals were identified based on fixed parameters in all images, and those within nuclei (defined by DAPI signal) were counted. Elaboration of the images was made by Adobe Photoshop CS 8.0 and statistical analyses were performed with GraphPad Prism 8; specific tests are indicated in Figure legends.

### 4.8. Immunofluorescence

Immunofluorescence was performed on cells grown on coverslips and fixed with cold methanol (100%) for 6 min at −20 °C. Blocking and incubations with both primary and fluorescently labelled secondary antibodies were performed at room temperature in PBS containing 0.05% Tween20 and 3% BSA. Cells were counterstained DAPI as described above. The primary antibodies used were rabbit anti-pT288 Aurora-A (3079S, Cell Signaling, 1:200) and mouse anti-γ-tubulin (T6557, Sigma Aldrich, 1:300). The fluorescent labeled secondary antibodies were anti-mouse FITC and anti-rabbit Cy3 (Jackson Immunoresearch, Cambridge House, UK). Images were acquired using a Nikon Eclipse 90i microscope equipped with a 100× (oil immersion; N.A. 1.3) objective and a Qicam Fast 1394 CCD camera (QImaging), using NIS-Elements AR 3.2 (Nikon) software; elaboration and processing were performed using NIS-Elements HC 5.11 (Nikon) and Adobe Photoshop CS 8.0. Quantitative measures of fluorescence intensity on the single centrosome (the mask for the analysis was performed on the signal of γ-tubulin) were performed using NIS-Elements HC 5.11 (nd2 file format), subtracting the internal background of each cell. Statistical analyses were performed with GraphPad Prism 8; specific tests are indicated in Figure legends.

### 4.9. Western Blotting 

For Western Blot analysis, synchronized or asynchronously growing cells were lysed in a RIPA buffer (50 mM Tris-HCl pH 8.0, 150 mM NaCl, 1% NP-40, 1 mM EGTA, 1 mM EDTA, 0.25% sodium deoxycholate) supplemented with protease and phosphatase inhibitors (Roche Diagnostic, Basel, Switzerland). Proteins were resolved by 10% SDS-PAGE and transferred on a nitrocellulose membrane (Protran BA83, GE Healthcare) using a semi-dry system (Bio-Rad). About 30 µg of extract per lane was loaded. The antibodies were mouse anti-N-Myc (NCM II 100, 1:100, Calbiochem), rabbit anti-Lamin B1 (ab16048, 1:2000 Abcam, Cambridge, UK), anti-mouse GAPDH (sc-32233, 1:2000, Santa Cruz Biotechnology) and rabbit anti-PARP (9542, 1:1000 o.n., Cell Signaling). HRP-conjugated secondary antibodies (BioRad) were revealed using the Clarity Western ECL substrate(BioRad, 170–5061). Where indicated, quantitative analysis on scanned films was performed using Adobe Photoshop 8.0. Signals were normalized for the loading control in each lane and the ratio with respect to the control lane (DMSO) was calculated. 

### 4.10. FACS Analysis

To analyze the cell-cycle phases distribution, both non-adherent and adherent cells were collected by centrifugation, washed with PBS, and fixed with 50% methanol overnight at 4 °C. Cell-cycle phase distribution was analyzed after incubation for 30 min in the dark with propidium iodide (PI, 0.03 mg/mL) and RNAsi A (0.2 mg/mL) using a flow cytofluorimeter Epics XL apparatus (Beckman Coulter, Brea, CA, USA). Cell aggregates were gated out on a bi-parametric graph FL-3lin/ratio as described in [47]. Cell samples were analyzed in a Coulter Epics XL cytofluorometer (Beckman Coulter) equipped with EXPO 32 ADC software. At least 10,000 cells per sample were acquired. The percentage of cells in the different phases of cell-cycle and in the sub-G1 compartment was calculated using Flowing Software 2.5.1. 

For the analysis of apoptotic cell death, after incubation with AnnexinV-FITC (IK-11120, Immunofluorescence Science Rome, Italy), either alone or in combination with propidium iodide (PI, Sigma-Aldrich P4170), Annexin V-FITC binding was analyzed via flow cytometry, using both flow cytometry FL-1 (FITC) and FL-3 (PI), acquired in a log amplification scale. Cell samples were analyzed in a FACSCalibur (BD Biosciences San Jose, USA), equipped with CellQuest software. At least 10,000 cells per sample were acquired. Data were processed using Flowing software 2.

## 5. Conclusions

Compared to other CD-inhibitors, the highly promising behavior of PHA-680626, together with its favorable drug-likeness, urge the in-depth investigation of this compound in the treatment of neuroblastoma. As discussed above, although the structural analysis of the highly similar Danusertib suggests the latter is unable to exert the same effects of PHA-680626, it will nevertheless be interesting to also investigate in future studies whether such “amphostericity” is a common attribute of this class of compounds. The recent report of successful combined use of AURKA CD inhibitors and ATR inhibitors in MYCN amplified neuroblastoma [48] strengthens the potential value of using compounds disrupting both AURKA activity and the interaction with N-Myc for the treatment of this highly aggressive tumor type.

## Figures and Tables

**Table 1:** List of AURKA inhibitors investigated in this study *.

PDB Code	Inhibitor	PDB ID	Selectivity	IC_50_ (nM)	Angle (°)
4J8M	CD532	CJ5	AURKA	35	92.9
2X81	MLN8054	ZZL	AURKA	6	84.6
2J4Z	PHA-680626	626	PAN	99	88.9
3UOH	RPM1722	0C4	AURKA	19	85.5
-	MLN8237 *	A5B	AURKA	6	85.0 **

* Alisertib (IC_50_ = 6 nM), whose structure in a complex with AURKA has not been solved yet, was also added for its high similarity with MLN8054. ** Value computed from the homology model based on 2X81.

**Table 2:** SPR analyses of ligands binding. Full kinetic analysis of the AURKA inhibitors.

Inhibitors	K_on_(M^−1^s^−1^) × 10^6^	K_off_(s^−1^) × 10^−3^	K_d_(nM)	Res SD	K_d_ Myc-AIR(μM)
CD532	3.58 ± 0.05	93.7 ± 0.70	26.2 ± 0.4	0.83	No binding
MLN8054	20.2 ± 0.03	46.1 ± 0.40	2.28 ± 0.03	1.254	No binding
PHA-680626	0.91 ± 0.04	7.83 ± 0.02	8.56 ± 0.04	3.7	No binding
RPM1722	2.61 ± 0.06	13.0 ± 0.10	5.0 ± 0.2	3	No binding
Alisertib	0.082 ± 0.1	5.04 ± 0.04	60 ± 1.0	2.3	48
MK8754	7.8 ± 0.1	12.0 ± 0.09	1.55 ± 0.03	1.65	0.7
ZM447439	0.096 ± 0.06	9.07 ± 0.04	92 ± 0.7	1.09	4

## Data Availability

Not applicable.

## Supplementary Materials

The following are available online at https://www.mdpi.com/article/10.3390/ijms222313122/s1.
